# Mutation of the H12-helix of α-tubulin/MEC-12 disrupts the localization of neuronal mitochondria

**DOI:** 10.17912/micropub.biology.000659

**Published:** 2022-10-23

**Authors:** Jean-Sébastien Teoh, Samiksha Dhananjay, Brent Neumann

**Affiliations:** 1 Neuroscience Program, Biomedicine Discovery Institute and Department of Anatomy and Developmental Biology, Monash University, Melbourne VIC 3800 Australia.

## Abstract

Microtubules are essential components of the cytoskeleton that allow bi-lateral neuronal transport. Correct regulation of these complex intracellular transport processes is central to neuronal function. However, despite major advancements in our knowledge, we still lack a complete understanding on how neuronal transport is regulated. Here, we provide further evidence for the importance of the highly conserved N-terminal H12-helix of α-tubulin. We show that a mutation in this region results in the mistargeting of axonal mitochondria in *Caenorhabditis elegans* , thereby establishing the importance of the H12-helix in regulating mitochondrial transport in neurons.

**Figure 1. Mitochondrial number and localization are disrupted in animals carrying the mec-12(u63) allele.**
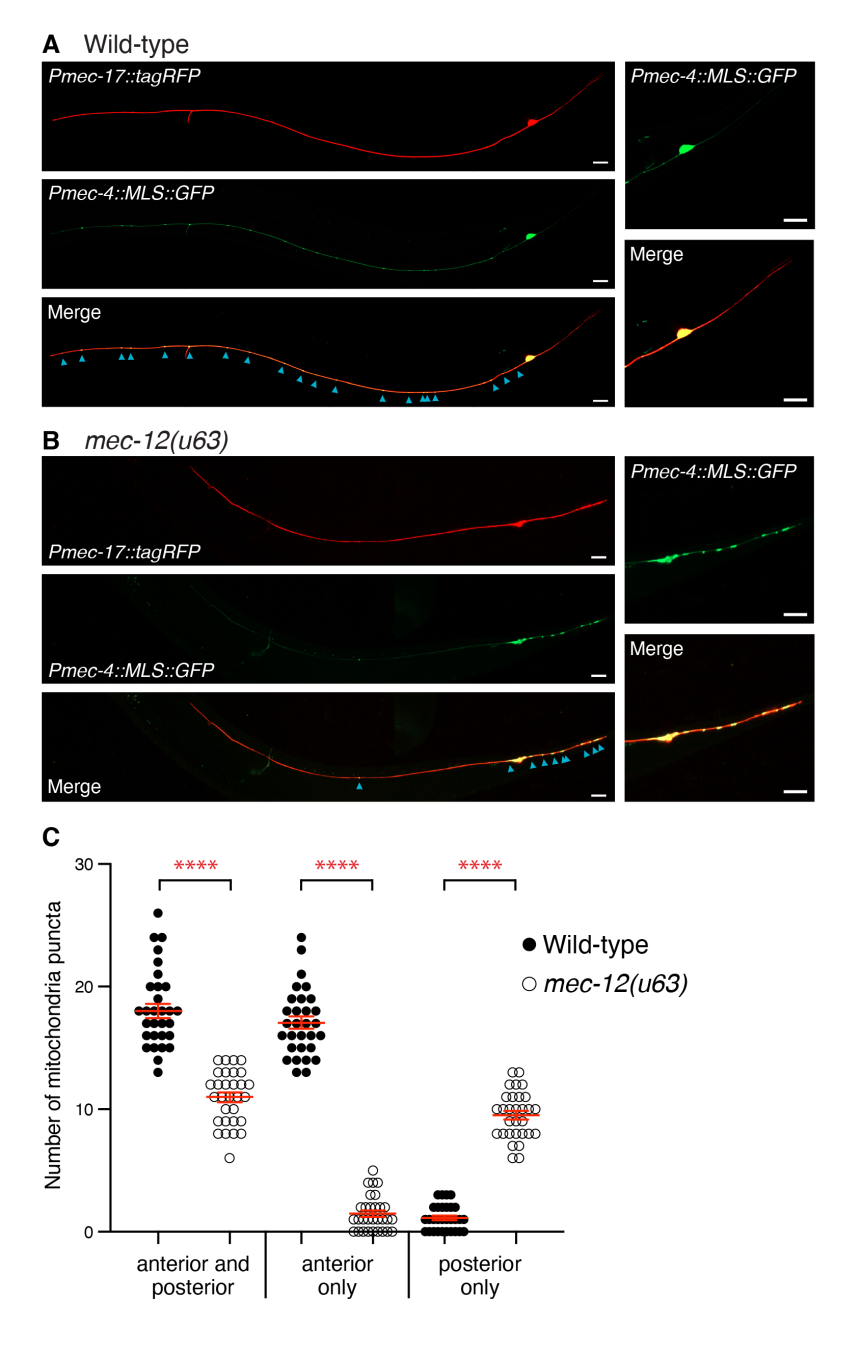
( **A** ) Confocal microscopy images of PLM neurons in a wild-type animal and ( **B** ) in a *mec-12(u63)* mutant. Top panels show the neurons diffusely labelled with tagRFP; middle panels show mitochondria puncta in the neurons labelled with GFP; bottom panels show a merged image of the tagRFP and GFP channels. Panels to the right show enlarged images of the posterior PLM neurites. Blue arrowheads in the merged panels highlight individual mitochondria puncta within the PLM neurites; scale bars = 10 µm. ( **C** ) Quantification of the number of mitochondria puncta in both the anterior and posterior PLM neurites, the anterior neurite only, or the posterior neurite only in wild-type and *mec-12(u63)* mutant animals. Symbols represent the values for individual animals; error bars represent mean ± standard error; P values calculated using one-way ANOVA with Tukey’s multiple comparisons test; **** P < 0.0001.

## Description

The three main components of a neuron (dendrites, soma, and axon) differ in both cellular structure and function (Kueh and Mitchison 2009, Akhmanova and Steinmetz 2015, Brouhard and Rice 2018). Many proteins that are synthesized in the soma are transported in a tightly regulated fashion to reach their required destination in the dendrites or axons. Microtubules form the structural network that allows and regulates this polarized transport of neuronal proteins (Kueh and Mitchison 2009, Akhmanova and Steinmetz 2015). Microtubules assemble from α- and β-tubulin heterodimers to form hollow cylinders that are subjected to post-translational modification and interaction with a variety of microtubule associated proteins (MAPs) (Desai and Mitchison 1997, Wloga and Gaertig 2010). Previous studies have suggested that molecular motor proteins including kinesin and dynein share a highly conserved and overlapping regulatory region at the C-terminus of α-tubulin, the H12-helix (Mizuno, Toba et al. 2004, Kikkawa and Hirokawa 2006, Tischfield, Baris et al. 2010, Redwine, Hernandez-Lopez et al. 2012, Niwa, Takahashi et al. 2013, Hsu, Chen et al. 2014, Uchimura, Fujii et al. 2015). The N-terminus of this highly conserved helix consists of acidic residues (414-417: EEGE) that are important for the interaction between kinesin, dynein, and microtubules (Hsu, Chen et al. 2014). Here, we studied the function of the H12-helix in regulating mitochondrial localization in the posterior lateral microtubule (PLM) neurons of *Caenorhabditis elegans* .

We visualized mitochondria in individual PLM neurons using the integrated transgene *jsIs609(Pmec-4::MLS::GFP)* (Mondal, Ahlawat et al. 2012). To investigate the importance of the H12-helix of a-tubulin, we analysed animals carrying the *u63* allele (E415K) of *mec-12/a-tubulin* , in which the glutamic acid (E) residue at position 415 is substituted with lysine (K) (Fukushige, Siddiqui et al. 1999). In contrast to wild-type animals, which displayed mitochondria puncta throughout the anterior PLM neurite (Fig. 1A, C), *mec-12(u63)* animals had almost no mitochondria puncta in these neurites (Fig. 1B, C). Instead, *mec-12* mutants had a large increase in the number of mitochondria puncta in the PLM posterior neurite compared to wild-type animals (Fig. 1C), suggesting that mitochondria are mistargeted in animals carrying the *mec-12(u63)* allele (Hsu, Chen et al. 2014). In addition, animals carrying the *mec-12(u63)* allele displayed significantly reduced overall numbers of mitochondrial puncta compared to wild-type controls within the PLM neurite (Fig. 1C, anterior and posterior data). Together, these results indicate the importance of the highly conserved α-tubulin H12-helix in regulating mitochondrial number and transport in the PLM neurons.

## Methods

***C. elegans* strains and genetics.** Animal manipulations were performed via standard procedures (Brenner 1974). Hermaphrodites were used for all experiments and were grown at 20 °C on nematode growth medium (NGM) plates seeded with OP50 *E. coli* . The *mec-12(u63)* mutant strain was used together with the following transgenes: *zdIs5(Pmec-4::GFP)* , *jsIs609(Pmec-4::MLS::GFP)* (Mondal, Ahlawat et al. 2012), and *uIs115(Pmec-17::tagRFP)* (Zheng, Jin et al. 2015).

**Analysis of mitochondrial localization** . Animals were immobilized in 0.05% tetramisole hydrochloride on 4% agar pads and imaged using a Zeiss Axio Imager M2 microscope with an Axiocam 506 mono camera and ZEN pro software. Mitochondrial puncta were visualized with the *jsIs609(Pmec-4::MLS::GFP)* transgene and quantified by manual counting. Representative images shown in Figure 1 were taken using a Zeiss LSM980 with Airyscan 2 confocal microscope (Objective Plan Apochromat 40x/1.3) equipped with ZEN 2 software. All images were taken using the Airyscan Multiples (MPLX)-Super Resolution (SR)-4Y mode. Bidirectional confocal imaging with 2x averaging was performed using a 488 nm solid state laser (11.0% power) and a 561 nm laser (2.4% power).

**Statistical analysis** was performed using GraphPad Prism 9. Statistical comparisons were made using one-way ANOVA with Tukey’s multiple comparisons test.
